# Metabolic Rates of Japanese Anchovy (*Engraulis japonicus*) during Early Development Using a Novel Modified Respirometry Method

**DOI:** 10.3390/ani13061035

**Published:** 2023-03-12

**Authors:** Dong In Kim

**Affiliations:** Aquaculture Research Institute, Kindai University, Shirahama 3153, Nishimuro, Wakayama 649-2211, Japan; donginkim0508@gmail.com or dongin_kim@kindai.ac.jp

**Keywords:** fish metabolism, allometry, oxygen consumption, pelagic fish, size-scaling metabolism, semi-closed method, growth, respirometry

## Abstract

**Simple Summary:**

Ontogenetic phase shift (i.e., a sudden change) in metabolism has been observed in several fish species during their early development, and understanding such metabolic shifts helps to understand their development trajectory. However, it is still unclear whether this sudden change in metabolism occurs typically in various fish species since larval-stage fish are vulnerable to physical stimuli due to their small size. Here, I developed a novel modified respirometry method by combining a peralstatic pump and the traditional semi-closed measurement method to measure metabolic rate changes in a pelagic fish species, the Japanese anchovy (*Engraulis japonica*). As a result, sudden changes in metabolism occurred in this species at a body weight of approximately 0.001 and 0.01 g. Furthermore, these changes coincided with their morphological and behavioral changes in this species. These findings confirm that a pelagic species undergoes similar metabolic changes during early development as the more widely studied non-pelagic species and will be useful in future metabolic studies.

**Abstract:**

The allometric relationship between metabolic rate (${VO}_{2}$) and body mass (M) has been a subject of fascination and controversy for decades. Nevertheless, little is known about intraspecific size-scaling metabolism in marine animals such as teleost fish. The Japanese anchovy *Engraulis japonicus* is a planktotrophic pelagic fish with a rapid growth and metabolic rate. However, metabolic rate measurements are difficult in this species due to their extremely small body size after hatching. Herein, the metabolic rate of this species during its early developmental stage was measured for 47 individuals weighing 0.00009–0.09 g (from just after hatching to 43 days old) using the micro-semi-closed method, a newly modified method for monitoring metabolism developed specifically for this study. As a result, three distinct allometric phases were identified. During these phases, two stepwise increases in scaling constants occurred at around 0.001 and 0.01 g, although the scaling exponent constant remained unchanged in each phase ($\hat{b}$ = 0.683). Behavioral and morphological changes accompanied the stepwise increases in scaling constants. Although this novel modified respirometry method requires further validation, it is expected that this study will be useful for future metabolic ecology research in fish to determine metabolism and survival strategy.

## 1. Introduction

In living organisms, metabolism is defined as the series of chemical reactions that convert ingested nutrients to the energy necessary for activity that culminates in the excretion of excess materials from the body. Simply put, metabolism is the exchange of energy between organisms and the environment. Metabolism is known to be involved in optimal resource allocation to ensure the maintenance and growth of living organisms and is closely related to the life cycle of each species [1,2,3]. The metabolic rate of living organisms can often be expressed as a power function of body weight (*M*) following the allometric equation ${VO}_{2}={aM}^{b}$ based on oxygen consumption (${VO}_{2}$), where a is the scaling constant and b is the scaling exponent [4,5]. Interestingly, many living organisms are negative scaled using this equation because their ${VO}_{2}$ has a scaling exponent of <1 [5,6,7,8,9,10,11,12]. This phenomenon has also been confirmed in inter- and intraspecies studies [5,6,7,8,10,11,13,14,15]. This relatively simple allometric equation has a limited ability to explain metabolic rate since it often cannot explain non-linear or curved log–log metabolic scaling relations [14,16,17,18]. Furthermore, the change in metabolic rate as an individual grows within a species is considered highly complex due to the many stages into which it can be broken down in an individual’s life [14,19,20,21,22,23,24]. This complexity further limits the ability of the simple allometric equation to explain the metabolic rate [2,14,19,21,22,23,24,25].

Intraspecies metabolism research is generally more advantageous than interspecies research when considering the complex changes in metabolism associated with body weight and size increases since many species display a large increase in these parameters during development [2,7,14,19,21,22,25,26,27,28,29,30]. The faster the increase, the easier it is to understand complex metabolic changes. Thus far, such intraspecies metabolism research has focused on invertebrates. Fish have been of particular interest because unlike mammals, birds, and reptiles, they develop from extremely small organisms during the larval stage (e.g., a weight of less than a few mg) and undergo a period of rapid growth, wherein their bodyweight and size significantly increase and can grow up to several hundred kilograms in their adult stage [21,22,25,26,27,31,32,33]. Therefore, fish are extremely suitable for studying intraspecies metabolism.

The scaling exponent has recently been found to be quite variable, with a range of 0.4 to 1.3, according to a meta-analysis of 138 studies of resting metabolism in 69 teleost species (representing 28 families and 12 orders) [34,35]. In fact, it has been reported that the early development stage (i.e., the larval period) tends to have an isometric or near isometric scaling exponent of b ≒ 1 [14,21,22,25,27,28,29,30,31]. In contrast, the allometric relationship between metabolic rate and body weight has been reported to become negatively allometric, with a simple regression of metabolic rate over body weight tending to be b < 1 [21,22,24,25,26,27,28,29,36,37,38,39]. However, it is currently unknown whether this phenomenon is generalized for intraspecies development due to a lack of empirical research (e.g., on ontogenetic phase shift in metabolism) to support the potentially isometric or near isometric state of the scaling exponent and due to the consideration of the various factors that can affect metabolic changes during early development [22,25].

The relationship between resting routine metabolic rate and body weight during the development of the tiger puffer (*Takifugu rubripes*) from hatching to juvenile stages was recently studied [21]. Interestingly, the relationship between oxygen consumption (${VO}_{2}$)—which is an indicator of metabolism according to ontogeny—and body weight (M) turned out to be ${VO}_{2}=a_{i}M^{0.795}$ [21]. Thus, it was clearly confirmed that the scaling constant increased in three steps (i.e., i = 1–4) [21]. In other words, the tiger puffer undergoes four ontogenetic phase shifts in their metabolism during development. In addition, the scaling exponent of overall metabolic rate over development (0.948 ± 0.002; estimate ± standard error of the mean) tended to be higher than each scaling exponent of the metabolic rates maintained during each stage (0.795 ± 0.019) [21]. These findings indicate that the metabolic rate per unit body weight decreased as body weight increased during early development, even though the overall energy metabolic rate remained high through the multiple increases of the scaling constant [21]. This suggests that the ontogenetic phase shift in metabolism may interact to affect the isometric or near-isometric scaling exponent in the tiger puffer [21]. In addition, the elevation of the scaling constant in tiger puffer studies provides clues about the relationship between morphology, behavior, and metabolism [21]. For example, it has been confirmed that cannibalism in tiger puffers peaks during these phase shifts, with the predators having undergone an elevation of the scaling constant and the prey having been unable to undergo the scaling constant elevation due to slow growth [21]. It can be inferred that morphologically and behaviorally more developed individuals acquire rapid motility that results in an advantage for survival compared to those that are less developed [40]. These findings are significant areas of research that can help us understand the relationships between intraspecies physiology, morphology, and behavior. However, additional empirical research is needed to determine whether these ontogenetic phase shifts in metabolism via stepwise elevations in scaling constants occur generally in different fish species since larval-stage fish are vulnerable to physical stimuli due to their small size.

A respirometry system, which is a system that measures the oxygen consumption in fish and thus metabolism in living conditions, includes the traditional Winkler titration method, which can be largely divided into closed, semi-closed, and constant flow (or open) systems [27]. Recently, polarographic, galvanic, or optical oxygen sensors that can connect these classical methods with electronic devices to measure metabolism have been mainly used [41]. Although it is certainly true that the accuracy of the measurement has been greatly improved by these remarkable technological advances in various systems and measurement technologies, underestimation of the oxygen consumption or measurement variability still occurs due to the signal noise inherent in electronic devices [42]. Nevertheless, this system that is based on an oxygen sensor is certainly a widely used method for research using metabolism measurement [43] because it enables continuous metabolism measurement, and its cost is inexpensive compared to those in the past (e.g., Strathkelvin Instruments). However, it is expected that it will take a considerable amount of time before it becomes popular among researchers with economic difficulties due to limited research funding, including those in developing countries. Furthermore, most detection instruments were frequently designed for relatively larger fish in the juvenile or adult stages with relatively higher body weights that are several grams or more [27]. In this study, a micro-semi-closed respirometry method was developed by using a peristaltic pump to solve the problems of the traditional Winkler titration methods, including closed, semi-closed, and constant flow methods, which can enable researchers with financial limitations to easily conduct metabolism measurement research. The approach of this current study addresses the previous inconsistent supply problem in the flow rate of water before the respirometry measurements, which had frequently occurred in the current Winkler titration method and was improved by supplying only a constant flow rate to the respiration chamber using a peristaltic pump. This improved the stability of the metabolic rate determination based on the discrepancy between dissolved oxygen concentration in the initial or inflowing water before the respirometry measurement and in the final or outflowing water after the measurement.

I tested this newly modified respirometry method on the Japanese anchovy (*Engraulis japonicus*), a species with significant and rapid body weight and size increases. Japanese anchovies are very small upon hatching (wet body weight; 0.00009 g) and highly vulnerable to the physical stimulation of minor handling. Currently, there are no known metabolic studies that focus on the early development of this species. In addition, it remains unknown whether the ontogenetic phase shift discussed earlier also appears in pelagic fish, such as the Japanese anchovy. The aim of this study was to use a micro-semi-closed method equipped with a peristaltic pump to measure the metabolic rate of the Japanese anchovy during early development and to use these results to explore the relationships between metabolic rate, morphology, and behavior during the early development of this species.

## 2. Materials and Methods

### 2.1. Fish Preparation

#### 2.1.1. Rearing Adult Japanese Anchovies

About 700 adult Japanese anchovies were bought from a fishing company (Takeshita Suisan, Nagasaki, Japan) and transported by truck in a 1000 L polycarbonate cylinder tank to the Fisheries Research Laboratory, Kyushu University, Fukuoka, Japan. The fish were divided into three round concrete tanks of 3000 L each and fed artificial diets three times daily. Naturally spawned *E. japonicus* eggs were collected during the night and transferred to polycarbonate cylinder tanks (200 and 500 L in size). The water temperature ranged between 20–25 °C. Salinity was maintained at 32–33 ppt during rearing. No artificial illumination was used during the experiment.

#### 2.1.2. Rearing Larvae and Juvenile Japanese Anchovies

One day after hatching, the mouth was opened and the yolk sac was partly absorbed. Two days after hatching, the yolk was completely absorbed, and the mouth became functional at temperatures of 23–25 °C [44]. After three days, the larvae were fed cultured rotifers (*Brachionus roduntiformis*) three times daily. After two weeks, newly hatched brine shrimp (*Artemia salina* L.) fortified with essential fatty acids, eicosapentaenoic acid (EPA), and docosahexaenoic acid (DHA) before feeding were fed twice daily. Fish used for the respirometry experiments were not fed for a period of 3–24 h before the experiments occurred, depending on developmental stage. Water temperature was maintained at 24–25 °C. All experimental processes were conducted according to the guidelines of Animal Experiments in the Faculty of Agriculture in Kyushu University, Fukuoka, Japan. This study was conducted in accordance with the suggestions offered in the ARRIVE guidelines (https://arriveguidelines.org; accessed on 22 December 2022).

### 2.2. Respirometry

**Figure 1:**
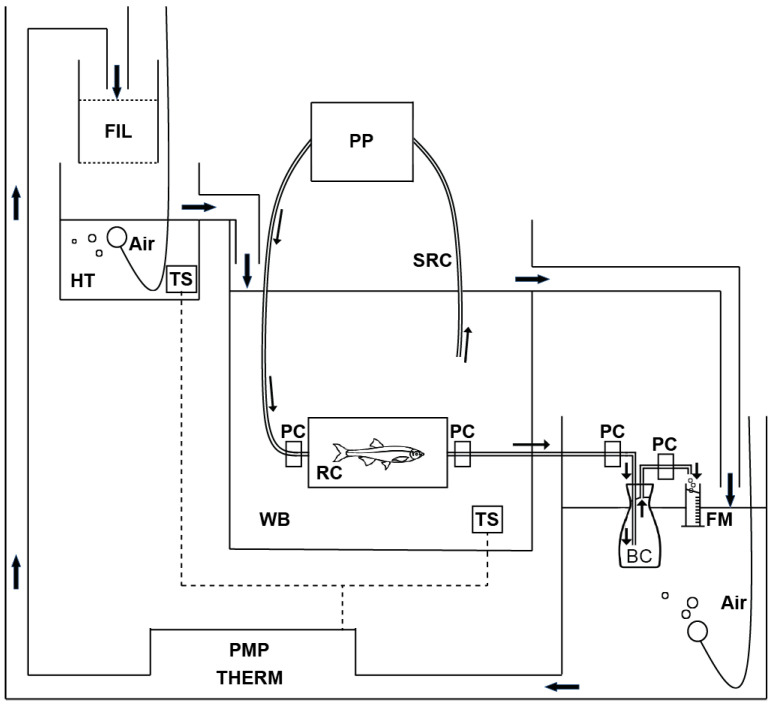
Diagram showing the micro-semi-closed method equipped with the peristalic pump. BC, blank changer; FIL, filter; FM, flow meter; HT, head tank; PC, pinchcock to close the RC and BC; PMP, pump; PP, peristaltic pumps; RC, respiration chamber; SRT, silicone rubber tube; THERM, thermostat; TS, temperature sensor; WB, water bath.

A total of 47 Japanese anchovies ranging in wet body weight from 0.00009 g (just after hatching) to 0.09 g (43 days old) were chosen for oxygen consumption measurement as a proxy for metabolic rate. Fasted larvae to early juveniles kept at 25 °C were examined for routine rates of oxygen consumption using a micro-semi-closed method (Figure 1). This method combined a conventional semi-closed method [27] with a peristaltic pump (AC-2110II, ATTO Corporation, Tokyo, Japan) (Figure 1). The detailed steps for respiration measurement used in this study are provided below.

#### 2.2.1. Process 1. Selection of the Sample and Acclimation in Respiration Chamber

Prior to measuring oxygen consumption, all fish were handled carefully since they were very susceptible to external stimuli, particularly during early development. Small beakers were used to collect the fish from the rearing tanks to avoid direct handling and to ensure that a sufficient number of fish were selected. Any dead or weak fish were quickly removed from the beaker using a pipette, as were any fish judged to be of a considerably different body size compared to the other fish. Next, the remaining fish were carefully transferred into 300 mL beakers. This procedure was performed multiple times until a sufficient number of fish were collected. The fish that seemed healthy were left for 1–3 h. After this period, any additional weak or dead fish were also removed using a pipette. The remaining fish were then placed in a respiration chamber. This approach helped to ensure an approximately 100% survival rate during measurement.

#### 2.2.2. Process 2. Control of the Water Flow in the Respiration Chamber Using a Peristaltic Pump

A peristaltic pump (AC-2110II, ATTO Corporation, Tokyo, Japan) was connected to the respiration chamber using a customized silicone rubber tube (3 mm in inner diameter and 5 mm in outer diameter; ATTO Corporation, Tokyo, Japan) that enabled a constant quantity of water to flow into the chamber. After that, a specially designed small bottle (approximately 20 mL) made from borosilicate glass was used to receive water from the respiration chamber. This bottle is also referred to as a blank chamber and was used in the next procedure.

#### 2.2.3. Process 3. Determination of Rate of Oxygen Consumption

To determine how much oxygen the fish consumed, the bottle (i.e., the blank chamber) was first completely sealed, then placed in a water bath (i.e., the respiration chamber). The dissolved oxygen concentration of the bottle was determined at the end of the experiment. If small air bubbles were observed in the respiration chamber or the blank chamber during this procedure, they were eliminated by infusing water from the outside using a pipette. The dissolved oxygen concentration of the blank chamber was used as the control because this was the initial oxygen concentration in the respiration chamber before the fish consumed any oxygen. When both chambers were sealed simultaneously, the dissolved oxygen concentration in the respiration chamber was used for the treatment group, since the fishes could consume this oxygen. The measurement time period after sealing was determined by the volume of the respiration chamber and the body size and number of fish. This procedure was carried out after extensive preliminary experiments were performed. Finally, the oxygen consumption was assessed by measuring the difference in the dissolved oxygen concentration between the control and the treatment groups. The Winkler titration method was used to calculate these two dissolved oxygen concentrations [27,45]. The micro-semi-closed method employed in this study is summarized in Table 1.

### 2.3. Statistics

Previously published static models were used, as provided below [21]. Multiple negative allometric relationships were investigated using two statistical models, excluding transitional phases. The first model was defined as

(1) $${VO}_{2}=a_{i}M^{\hat{b}}$$

where $a_{i}$ is an intragroup scaling constant of the ith group, and $\hat{b}$ is the scaling exponent of each group. Equation (1) was used to represent each incidence of negative allometry. The second model was defined as

(2) $${VO}_{2}=\alpha M^{\bar{b}}$$

where α is an intergroup of one of the groups of fish, and $\bar{b}$ is the overall scaling exponent. Equation (2) was used to determine how these allometries are related to the overall (i.e., intergroup) line. These two equations can be recast as the following equations:

(3) $$yij=loga_{i}+\hat{b}xij+\varepsilon ij$$

and

(4) $$yij=log\alpha +\bar{b}xij+Eij,$$

where yij is $log{VO}_{2}$; xij is logM; and εij and Eij represent the random variations in metabolism of the intra-and intergroups, respectively. The sum of the µi squares, that is, the vertical distance of the group means (${\bar{x}}_{i},{\bar{y}}_{i}$) from the overall line was minimized by estimating $\bar{b}$ and logα using ordinary least-squares regression [46]. Since logαi may be expressed as ($log\alpha +µi+\left(\bar{b}-\hat{b}\right){\bar{x}}_{i}$), the previous Equation (4) can be recast as the following equation [47]:

(5) $$yij=log\alpha +µi+\left(\bar{b}-\hat{b}\right){\bar{x}}_{i}+\hat{b}xij+\varepsilon ij$$

The degree of statistical significance was determined using the one-way analysis of covariance (ANCOVA), which included estimating the sums of squares, degrees of freedom, and mean squares for each term of Equation (5). Microsoft Excel for Office 365 was used for all data analyses (Microsoft Corp., Redmond, Washington, DC, USA).

## 3. Results

**Figure 2:**
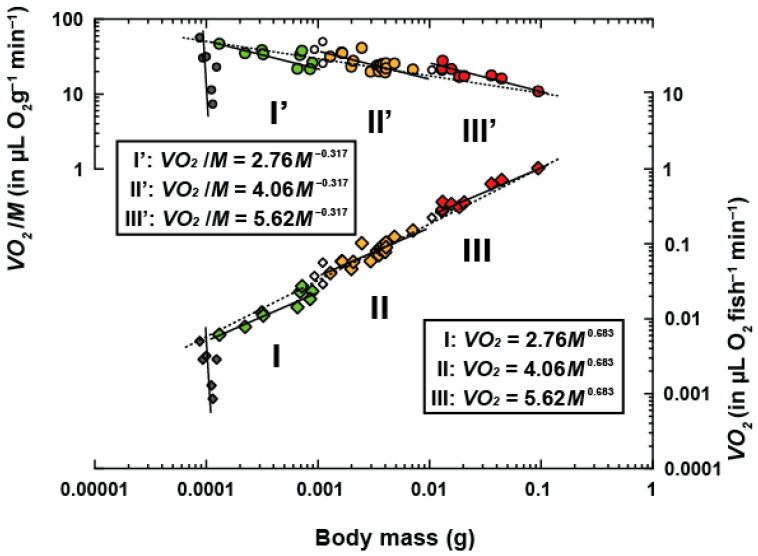
Ontogenetic changes in the relationship between the rate of respiration (${VO}_{2}$, diamonds) and body weigth (M) in the Japanese anchovy at 25 °C using the micro-semi-closed method. Ranges covered by the three solid black lines for ${VO}_{2}$ and ${VO}_{2}/M$ indicate intragroup phases of negative allometry. The vertical lines at around 0.0001 g represent ${VO}_{2}$, which increased daily from just after hatching to 43 days after hatching. Small open diamonds and circles represent values during the transitional phases.

The relationship between oxygen consumption rate (${VO}_{2}$ in µL $O_{2}$ fish^−1^ min^−1^) and body weight (M in g) are plotted in Figure 2. Mass-specific rates of oxygen consumption (${VO}_{2}/M$ in µL $O_{2}$ fish^−1^ min^−1^) are also presented. There was no noticeable allometric relationship between ${VO}_{2}$ and M during the early larval stage since the metabolic rates of the small larvae of a similar weight varied greatly. Thus, even though it seems to be linearly vertical on the log–log plot, the metabolism–body-weight relationship cannot be expressed as an allometric equation ${VO}_{2}=\alpha M^{b}.$ This straight upward distribution of metabolic rate values in ${VO}_{2}$ and ${VO}_{2}/M$ suggests that mass-specific metabolic rate rapidly increased during this stage (Figure 2). Thus, I did not perform the regression analysis of the mass–metabolism relationship for this stage.

A summary of the regression analysis for each intra- and intergroup is presented in Table 2. There are three negative allometric relationships between ${VO}_{2}$ and M from the 0.00013 g (6 days old) to the 0.09428 g (43 days old) fish, with two stepwise increases in the scaling constants ($a_{i}$ = 1–3) that occur at approximately 0.001 and 0.01 g of body weight and with a scaling exponent that was maintained during each phase ($\hat{b}$= 0.683). The scaling exponents of the individual lines ($\hat{b}$) were significantly smaller than the unity for all regression lines (*p* < 0.05; two-tailed *t*-test). There were no statistically significant differences among the three groups in terms of the slopes of the individual lines ($\hat{b};$ *F*_2,30_ = 0.09; *p* = 0.918; ANCOVA). The estimated value of the scaling exponent of the intragroup was confirmed to be $\hat{b}$ = 0.683 ± 0.049. The estimated values of the logarithm of the scaling constant for each group at $\hat{b}$ = 0.683 were $loga_{1}$ = 0.441 ± 0.167; $loga_{2}$ = 0.609 ± 0.125; and $loga_{3}$ = 0.749 ± 0.084. The scaling constants were estimated as $a_{1}$ = 2.76; $a_{2}$ = 4.06; and $a_{3}$ = 5.61. The interspecific scaling exponent was estimated to be $\bar{b}$ = 0.862 ± 0.003.

To verify the correctness of Equation (5), an ANCOVA was conducted (Table 3). No significant difference between the *µi* values and zero were found (*F*_1,32_ = 0.59; *p* = 0.447; ANCOVA). It is possible that the group means were placed on the overall line. Additionally, there was a statistically significant difference between the intragroup scaling exponent ($\hat{b}$ = 0.683) and the intergroup exponent ($\bar{b}$ = 0.862; *F*_1,32_ = 11.42; *p* = 1.93 × 10^−3^). Given these findings, it can be concluded that the intragroup scaling constant increased significantly from $a_{1}$ = 2.76 to $a_{3}$ = 5.61 with an increase in body weight (Figure 2). Thus, the ontogenetic phase shifts in metabolism that occurred with growth during early development represented by ${VO}_{2}=a_{i}M^{0.683}$ were due to two stepwise increases in ai at approximately 0.001 and 0.01 g, with each scaling exponent remaining constant during each phase.

## 4. Discussion

### 4.1. The Relationship between Metabolic Variation Patterns and Developmental Changes

In this study, Japanese anchovies ranging from 0.00009 to 0.09 g in wet body weight and from approximately 3 to 25 mm in body length from just after hatching to 43 days old during early development were studied. Three phases of metabolic scaling constants ($a_{1}$ = 2.76; $a_{2}$ = 4.06; and $a_{3}$ = 5.61, respectively; Figure 2 and Table 2) were identified. During the transitional phases, the scaling constant increased twice, at approximately 0.001 and approximately 0.01 g (Figure 2 and Table 2). In contrast, the scaling exponent was maintained in each phase ($\hat{b}$ = 0.683) (Figure 2). These results show for the first time that pelagic fish, such as Japanese anchovy, also undergo a metabolism shift during early development. This finding may have significant implications for other ecological and physiological processes, since this species is frequently preyed on by higher predators, such as mackerels, skipjacks, tunas, yellowtails, sea birds, marine mammals, and invertebrates [48,49,50,51,52,53,54]. Previous results suggest that the multiple stepwise increase in the metabolic scaling constant may be closely related to predator–prey interactions (i.e., cannibalism) in tiger puffer fish, although cannibalism is likely to have a higher impact in an aquarium than in nature [21]. This indicates that fast-growing individuals can rapidly increase their metabolic scaling constant and successfully shift to the next metabolic phase, thus enabling high anti-predator adaption [21]. In contrast, slow-growing individuals are deficient in a variety of morphological and/or behavioral characteristics; hence, they have a worse chance of survival since they cannot protect themselves against predators [21]. This explanation supports the growth–mortality hypothesis, which asserts that larger, more rapidly growing and developing individuals have a greater chance of survival [40]. Interestingly, it may also apply to pelagic fishes, such as the Japanese anchovy, because the ontogenetic shifting of their metabolic rate is significantly related to morphological and behavioral changes (Table 4).

During the transitional phase that occurred at approximately 0.001 g (18 days old), fin folds disappeared completely, the notochord started to flex, and the caudal fin ray was completed [44]. These changes could influence the swim capabilities and feed habits of the fish [55]. In fact, the fish larvae were successfully switched from feeding on rotifers (*Branchionus* sp.) to brine shrimp (*Artemia* sp.) during this stage, since their digestive systems were filled with orange-colored artemia, a condition known as artemia syndrome, a few hours after feeding (Table 4). This implies that their morphological changes are strongly related to their feed habits and swim capabilities. This study does not provide data regarding how their swimming abilities changed during the metabolic transitional phase, but it can be indirectly inferred through the swimming ability of the prey (i.e., rotifer and brine shrimp). Generally, the swimming speed of brine shrimp is approximately five times faster than that of rotifers (3.05 mm s^−1^ versus 0.62 mm s^−1^, respectively) [56]. Although this study was unable to provide a mechanism for the shift in feeding habits in this species due to the various complicated causes (i.e., visual, digestive system) involved, a comparison of the swimming speeds of the prey organisms implies that fast-growing individuals may have the ability to swim faster than slow-growing individuals. More importantly, these morphological and behavioral changes coincide with the transitional period, when the scaling constant dramatically increases (Table 4).

During the transitional phase that occurred at approximately 0.01 g (29 days old), the larvae were considered to have attained the juvenile stage due to their morphological traits, including fully developed fins, rays, and pectoral fins [44,57]. Similar to the transitional phase at approximately 0.001 g, such a morphological change may have significant effects on behavior (Table 4). In fact, Fukuhara [44] and Masuda [58] suggested that morphological changes during growth increased swim speed and abilities, which led to schooling behavior. Ohata [53] also reported that when the larvae reached the juvenile stage, their swim speed dramatically increased compared to that of the larvae. Thus, an increase in the scaling constant with growth implies that fast-growing individuals can begin schooling behavior earlier than slow-growing individuals, thus resulting in better predator avoidance. Even though there was an experimental limitation in the findings of this study owing to the limited number of individuals examined, these findings might be an essential step in the process of better understanding the eco-physiological traits of pelagic fishes. To clarify the relationship between the change patterns of these traits and changes in metabolism patterns, future studies should collect data quantifying continuously changing traits such as morphology and behavior, using the individuals used for metabolism measurements as subjects. In addition, the mechanism by which the scaling constant rapidly increases before and after the transitional phase will be investigated by a molecular genetics approach (e.g., a genome-wide analysis of mRNA expression and transcription activation related to metabolism).

**Table 4:** Morphological and behavioral characterization of Japanese anchovy in different phases of development.

Transitional Phase between (Wet Body Weight in g)	Day after Hatching	Morphological and Behavioral Characteristics
$a_{1}$ and $a_{2}$ (approx. 0.001)	18	Melanophores were distributed along the body surface. Fin folds disappeared completely, the notochord started to flex, and caudal fin ray was completed [44]. Larvae started to eat live brine shrimp and *Artemia* sp. and tended to have *Artemia* syndrome and became orange in color a few hours after feeding.
$a_{2}$ and $a_{3}$ (approx. 0.01)	29	Transformations to juvenile occur when fins and rays were fully developed [57] The pectoral fin rays were completed [44]. They had increasing the swimming speed with growth and began to show schooling behavior [44,53,58]. They were eating small particles of chopped meat of *Euphausia* sp. given as a food [44].

### 4.2. Consideration of Methodological Constraints and Proposals for Further Progress

The existing Winkler titration method adjusts water flow by connecting a valve to the input and output parts of the respiratory chamber [27], but adjusting water flow to a constant level using this method has been inconvenient. In addition, when measuring metabolism using small fish larvae at sizes such as 0.0001–0.001 g, a delicate control of the water flow (e.g., 2–10 mL min^−1^) is required (Table 1). However, this type of manually controlled water flow by using valves is difficult and complicates metabolism measurements in some cases because of the physical damage caused to larvae due to the large influx of water flow into the respiratory chamber. It also causes handling problems such as the death of larvae due to them being sucked into the pipe of the output part. In this study, a peristaltic pump was installed in the micro-semi-closed method, a method used to solve the various problems that arise in the process of manually controlling water flow using a valve in the existing Winkler titration method to conduct metabolism measurements on the Japanese anchovy, a pelagic fish. Using this method, patterns in ontogenetic phase shifts in metabolism, which have been reported in studies using the tiger puffer and Japanese flounder, have been observed in Japanese anchovy. Nevertheless, as described in Table 1, the measurement method used in this study also has limitations that arise due to conducting metabolism measurements using multiple individuals during the early larvae stage. That is, in larvae before 0.0006 g (15 days old), metabolism measurements were performed using multiple individuals. The Japanese anchovy, a pelagic fish, is generally known as a species that continuously performs swimming activities [44]. This characteristic may affect the accuracy of metabolism measurements when they are performed on multiple individuals, as the measurements may be influenced by the swimming activities among individuals. However, swimming activities were hardly observed (nearly stationary state) up to a week after hatching in the Japanese anchovy used for this study. For individuals before 0.0006 g, with which multiple individuals were used to carry out metabolism measurements, a relatively large respiratory chamber (i.e., 36–40 mL) compared to the size of the fish was used for metabolism measurements; thus, it is judged that the effect due to swimming activities among individuals is minute. In addition, since the transitional phase, during which the scaling constant increased sharply, was observed before and after 0.001 and 0.01 g when metabolism measurements were carried out using single individuals, there is no doubt regarding the possibility of an ontogenetic phase shift in metabolism in this species. Nevertheless, as metabolism measurements were carried out using multiple individuals in several milligrams of larvae immediately after hatching, the influence on the patterns of metabolism measurements due to the swimming activities among the individuals within the respiratory chamber must also be taken into consideration. As an alternative countermeasure to this problem, a future study with improved research methods using a respiratory chamber smaller in size is necessary to enable metabolism measurements for single individuals, including even those immediately after hatching that are extremely small and have a body mass of only several milligrams. Although the micro-semi-closed method used in this study requires further validation through comparison with other measurement methods, this method could be used in the future to measure the metabolism of various organisms based on the similarity between the findings reported here in a pelagic fish species and those of previous studies on non-pelagic fish. Future study should validate this novel modified respirometry method and further elucidate the elevations in the metabolic scaling constant during growth using biological and molecular approaches.

## 5. Conclusions

The present study determined the routine metabolic rate in a pelagic fish species, the Japanese anchovy, using a micro-semi-closed method, a newly modified respirometry measurement system. The results showed that ontogenetic phase shifts in metabolism occur in this pelagic fish species, a finding that had previously only been recorded in other fish species. Regression analysis of the relationship between metabolic rate and body weight depending on ontogeny revealed three distinct negatively allometric phases. The metabolic scaling constant tended to increase rapidly around 0.001 and 0.01 g, but the metabolic scaling exponent (i.e., for each group line) remained constant during each phase ($\hat{b}$ = 0.683). Interestingly, the timing of these rapid metabolic changes coincided with rapid changes in morphology and behavior. Although further validation of the respirometry measurement method in this study is necessary, it is expected that the current study will be useful for future metabolic ecology research in both pelagic fish and other fish species to better understand their metabolism and survival strategies.

## Figures and Tables

**Table 1:** Summary of respirometry system based on the micro-semi-closed method.

Range of Body Weight (g)	Volume of Respiration Chamber (mL)	No. of Fish	Respirometry Time (min)		Flow Rate of Water (mL min^−1^)	$O_{2}$ $\;\mathrm{in}\;\mathrm{mL}\cdot l^{-1}\;(\bar{x}\pm S.D.)$	
			before Measurement	during Measurement		Initial Inflow	Final Outflow
0.0001–0.002	36–40	41–1	60–90	120–540	2–10	4.67 ± 0.11 (23)	4.33 ± 0.22 (23)
0.003–0.02	45–60	1	90–120	90–270	12–20	4.82 ± 0.10 (20)	4.14 ± 0.36 (20)
0.03–0.04	240–260	1	180–210	200–220	23–31	4.76 ± 0.03 (2)	4.20 ± 0.02 (2)
0.05–0.1	260–340	1	210–240	180–200	31–52	4.89 ± 0.10 (2)	4.33 ± 0.11 (2)

**Table 2:** Intragroup (rows 1–3) and intergroup (rows 4–6) regression analyses for the relationship between $log{VO}_{2}$ (${VO}_{2}$ equals the total oxygen consumption in µL $O_{2}$ fish^−1^ min^−1^) and $log{VO}_{2}$ (*M*, body weight in g) using the micro-semi-closed method.

Group	*N*	Range of Body Weight (g)	Scaling Constant	$\mathrm{Scaling}\;\mathrm{Exponent}\;(\bar{x}\pm S.D.)$	*P*	*R^2^*
1 ^a^	9	0.00013–0.00085	3.38	0.709 ± 0.100	2.29 × 10^−2^	0.878
2 ^a^	18	0.00129–0.00709	4.02	0.682 ± 0.094	3.90 × 10^−3^	0.765
3 ^a^	9	0.01295–0.09428	5.12	0.659 ± 0.064	1.12 × 10^−3^	0.938
1–3 ^b^	36	0.00013–0.09428	α = 11.22	$\hat{b}$ = 0.862 ± 0.003	1.43 × 10^−34^	1.000
1–3 ^c^	36	0.00013–0.09428	9.79	0.839 ± 0.020	3.37 × 10^−9^	0.980
Total ^d^	47	0.00009–0.09428	14.34	0.911 ± 0.030	4.93 × 10^−3^	0.953

^a^ Parameters were estimated to minimize the sum of squares of squares of *ε_ij_* in each group; ^b^ parameters were estimated to minimize the sum of squares of *µ_i_*; ^c^ parameters were estimated to minimize the sum of squares of *Eij*; ^d^ including the transitional phases.

**Table 3:** One-way analysis of covariance outcomes to measure oxygen consumption in Japanese anchovies during the early development stages at 25 °C.

Term	Sum of Squares	Degrees of Freedom	Mean Square	Mean–Square Ratio	*P*
log α	44.2080678	1	44.208068	8978	8.98 × 10^−41^
*µ_i_*	0.00291609	1	0.002916	0.59	4.47 × 10^−1^
*(*$\bar{b}$ − $\hat{b}$ *)* $\bar{x}$ *i*	0.05621026	1	0.056210	11.42	1.93 × 10^−3^
$\hat{b}$ *x_ij_*	10.7264528	1	10.726453	2178	5.24 × 10^−31^
*ε_ij_*	0.15756394	32	0.004924		
Total (about mean)	10.9431431	35			
Total (about zero)	55.1512109	36			

## Data Availability

Data generated in this study are available on request from the corresponding author.
